# Author Correction: Mitochondria-specific drug release and reactive oxygen species burst induced by polyprodrug nanoreactors can enhance chemotherapy

**DOI:** 10.1038/s41467-019-10186-0

**Published:** 2019-06-10

**Authors:** Wenjia Zhang, Xianglong Hu, Qi Shen, Da Xing

**Affiliations:** 1 MOE Key Laboratory of Laser Life Science & Institute of Laser Life Science, South China Normal University, 510631 Guangzhou, China; 2 College of Biophotonics, South China Normal University, 510631 Guangzhou, China

**Keywords:** Cancer, Drug discovery, Cancer, Bioconjugate chemistry

Correction to: *Nature Communications* 10.1038/s41467-019-09566-3, published online 12 April 2019.

This Article contains an error in Fig. 7. In panel d, images depicting NT-PNs and TPP-PNs were inadvertently taken from the Control and cRGD-PNs images, respectively. The correct version of the Figure is shown below as Fig. 1.

**Figure 1:**
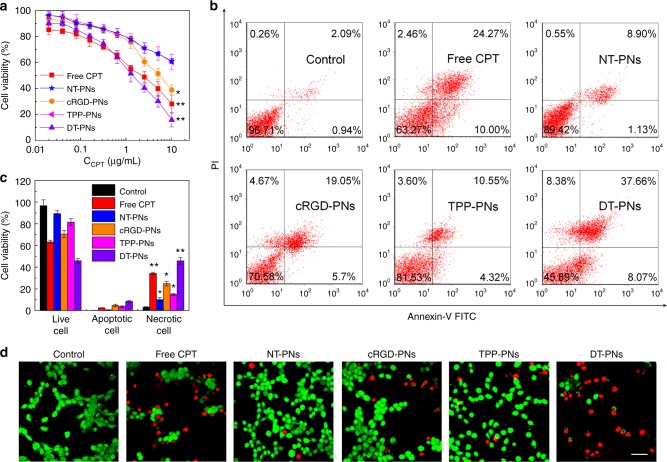
Fig. 1

