# Author Correction: Mitochondria-specific drug release and reactive oxygen species burst induced by polyprodrug nanoreactors can enhance chemotherapy

**DOI:** 10.1038/s41467-019-12198-2

**Published:** 2019-10-04

**Authors:** Wenjia Zhang, Xianglong Hu, Qi Shen, Da Xing

**Affiliations:** 1 MOE Key Laboratory of Laser Life Science & Institute of Laser Life Science, South China Normal University, 510631 Guangzhou, China; 2 College of Biophotonics, South China Normal University, 510631 Guangzhou, China

**Keywords:** Cancer, Drug discovery, Cancer, Bioconjugate chemistry

Correction to: *Nature Communications* 10.1038/s41467-019-09566-3, published online 10 June 2019.

This Article contained errors in Figure 8 and Supplementary Figure 11. In Figure 8 panel d, the image depicting 1 day DT-PNs treatment was inadvertently replaced with an additional image of the mouse shown in the panel illustrating 1 day PBS treatment. In Supplementary Figure 11c the image depicting cRGD-PNs was inadvertently replaced with an additional image of the cells shown in the panel illustrating Free CPT. The correct images of the mice and the cells are now provided in Figure 8d and Supplementary Information figure 11c, respectively. These errors have been corrected in the HTML and PDF versions of the Article.

