# Supplementary material for: Mitochondria-specific drug release and reactive oxygen species burst induced by polyprodrug nanoreactors can enhance chemotherapy
Source: Nat Commun. 2019 Apr 12;10:1704. doi: 10.1038/s41467-019-09566-3 (PMC6461692; doi:10.1038/s41467-019-09566-3)
Supplement: Supplementary file 1 — Supplementary Information [file 41467_2019_9566_MOESM1_ESM.pdf]

Supplementary Information

Mitochondria-specific polyprodrug nanoreactors for self-circulation drug release with reactive oxygen species burst in chemodynamic therapy

Zhang et al.

Supplementary Methods

Materials

4-bromobutyric acid ($C_4H_7BrO_2$, 98%), 2-hydroxyethyl methacrylate (HEMA, 99%), 1-Ethyl-3-[3-dimethylaminopropyl] carbodiimide hydrochloride (EDC), *N*-hydroxysuccinimide (NHS), (2-hydroxyethyl) triphenylphosphonium bromide (TPP), 3-(4,5-dimethylthiazol-2-yl)-2, 5-diphenyltetrazolium bromide (MTT), camptothecin (CPT), titanium tetrachloride ($TiCl_4$, 99.9%), potassium dioxide (KO_2) sodium nitrite ($NaNO_2$), sodium hypochlorite (NaOCl) and other chemicals were all purchased from Sigma-Aldrich. 2, 2'-azobis(2-methylpropionitrile) (AIBN) was obtained from Acros Chemicals and recrystallized from ethanol. Amine-functionalized cRGD was obtained from GL Biochem Ltd. MitoSox Red, MitoTracker Red and LysoTracker™ Green were purchased from Thermo Scientific. Calcein-AM was purchased from J&K Scientific, Beijing, China. Cellular Reactive Oxygen Species Detection Assay (Deep Red Fluorescence) was purchased from Abcam. MitoTracker Green, DCFH-DA, Propidium Iodide (PI), JC-1, Annexin V-FITC Apoptosis Detection Kit and ATP Assay Kit were purchased from Beyotime.

Synthesis of 4-mercapto-butyric acid

4-bromobutyric acid (5.0 g) and thiourea (2.4 g) were refluxed in ethanol (100 mL) for 4 h.[1,2] After addition of NaOH (12 g, in 150 mL of ethanol), the solution was refluxed overnight. After cooling, white precipitate was formed and isolated by vacuum suction filtration and washed with cold ethanol. Then resuspending the precipitate in 100 mL water, the aqueous phase was then acidified with 4 M HCl to pH 5, then extracted twice with an equal volume of diethyl ether. After drying with anhydrous Na_2SO_4, the organic layer was evaporated, affording 4-mercapto-butyric acid (1.61 g, 45% yield).

Synthesis of ROS-cleavable thioketal linker (TK)

Anhydrous acetone (0.7 mL) and 4-mercapto-butyric acid (1.5 g) were mixed in $CHCl_3$ (10 mL), then added with 0.3 equivalent $TiCl_4$ at -10 ℃.[3] Instantly, the solution turned yellowish formed with precipitate. The resulting reaction mixture was allowed to warm-up slowly to room temperature and stirred overnight. White precipitate was formed and washed thoroughly with diethyl ether. Finally, colorless solid was obtained and dried under reduced pressure to afford TK (1.24 g). ^{1}H NMR (300 MHz, $CDCl_3$) δ 2.68 (t, *J* = 7.2 Hz, 4H), 2.50 (t, *J* = 7.3 Hz, 4H), 1.90 (p, *J* = 7.2 Hz, 4H), 1.58 (s, 6H).

Synthesis of HEMA-TK

TK (1.0 g) and DMAP (0.49 g) in anhydrous DCM (30 mL) was added 2-hydroxyethyl methacrylate (HEMA) (0.52 g) at room temperature.[4] After stirring for 10 min, EDC (1.2 g) dissolved in anhydrous DCM (5 mL) was slowly added to the above solution under N_2 atmosphere. The reaction was performed under nitrogen atmosphere for 24 h at room temperature. After that, the reaction mixture was then extracted third with an equal volume of brine. The organic layer was separated, dried over anhydrous Na_2SO_4, filtered and concentrated on a rotary evaporator. The crude product was purified by column chromatography using an eluent of 1:1 hexane: ethyl acetate, yielding colorless solid. ^{1}H NMR (500 MHz, $CDCl_3$) δ 6.12 (td, *J* = 3.9, 1.9 Hz, 1H), 5.59 (td, *J* = 4.0, 2.0 Hz, 1H), 4.39 - 4.25 (m, 4H), 2.68 (t, *J* = 7.2 Hz, 4H), 2.50 (t, *J* = 7.3 Hz, 4H), 1.93 (m, 3H), 1.90 (p, *J* = 7.2 Hz, 4H), 1.58 (s, 6H).

Synthesis of ROS-cleavable CPT prodrug monomer (CPTSM)

Briefly, a mixture of CPT (0.5 g), HEMA-TK (0.6 g) and DMAP (0.18 g) were suspended in anhydrous DCM under N_2 atmosphere, then EDC (0.42 g) dissolved in anhydrous DCM (5 mL) was added slowly under N_2 atmosphere and stirring for 10 min at room temperature.[5] The reaction was performed under nitrogen atmosphere for 24 h at room temperature. After that, the reaction mixture was then extracted brine, and the organic layer was separated, dried over anhydrous Na_2SO_4, and concentrated

on a rotary evaporator. The solid residues were purified by column chromatography using an eluent gradient from the mixture of hexane and ethyl acetate to afford the resulting CPTSM (0.82 g). The chemical structure of CPTSM was verified by ^{1}H NMR and ^{13}C NMR analysis (Figure S1). ^{1}H NMR (500 MHz, $CDCl_3$) δ 8.40 (s, 1H), 8.22 (d, *J* = 8.5 Hz, 1H), 7.94 (d, *J* = 7.9 Hz, 1H), 7.88 – 7.80 (m, 1H), 7.70 – 7.63 (m, 1H), 7.22 (s, 1H), 6.12 (td, *J* = 3.9, 1.9 Hz, 1H), 5.68 (d, *J* = 17.2 Hz, 2H), 5.59 (td, *J* = 4.0, 2.0 Hz, 1H), 5.41 (d, *J* = 17.2 Hz, 2H), 4.39 – 4.25 (m, 4H), 2.68 (t, *J* = 7.2 Hz, 4H), 2.50 (t, *J* = 7.3 Hz, 4H), 1.93 (m, 3H), 1.90 (p, *J* = 7.2 Hz, 4H), 1.58 (s, 6H), 1.02 – 0.92 (m, 3H); ^{13}C NMR (101 MHz, $CDCl_3$) δ 174.18, 173.13, 171.96, 167.16, 158.34, 155.29, 147.40, 143.05, 137.66, 132.73, 130.28, 129.18, 128.39, 128.25, 127.93, 127.57, 124.72, 117.50, 96.46, 78.27, 63.28, 62.67, 61.25, 56.68, 50.71, 32.90, 32.70, 30.99, 30.45, 29.29, 26.07, 19.10, 7.85. APCI-MS: m/z calc. for $C_{37}H_{42}N_2O_9S_2$ 722.23; found 723.24 $[M+H]^+$.

Synthesis of TPP-conjugated chain transfer agent (CPPA-TPP)

Briefly, a mixture of 4-cyano-4-(phenylcarbonothioylthio) pentanoic acid (CPPA, 0.12 g), TPP (0.20 g) and DMAP (0.42 g) were suspended in 10 mL anhydrous DMF under N_2 atmosphere. EDC (0.13 g) dissolved in anhydrous DCM (5 mL) was added slowly to above solution under N_2 atmosphere, then stirring for 10 min at room temperature. The reaction was performed under nitrogen atmosphere for 12 h at room temperature. After that, the reaction mixture was concentrated on a rotary evaporator. The solid residues were purified by column chromatography using an eluent gradient from 100% EtOAc to 100% acetone to afford CPPA-TPP as an orange solid (0.22 g). ^{1}H NMR (500 MHz, $CDCl_3$) δ 8.07 – 7.30 (m, 20H), 5.30 (s, 2H), 2.66 – 2.56 (m, 2H), 1.78 – 1.47 (m, 2H), 1.25 (s, 3H); ^{13}C NMR (101 MHz, $CDCl_3$) δ 210.92, 168.82, 145.14, 144.23, 132.49, 130.83, 129.75, 128.49, 128.32, 124.65, 118.99, 69.66, 53.90, 31.78, 29.27.

Synthesis of hydrophilic PDMA

Reversible addition-fragmentation chain transfer (RAFT) polymerization was

employed for the synthesis of PDMA hydrophilic polymer.[6] Typically, CPPA-TPP (30 mg), DMA (639 mg), and AIBN (3.54 mg) were charged into a glass ampoule containing 1,4-dioxane (1.2 mL). The ampoule was then degassed via three freeze-pump-thaw cycles and flame-sealed under vacuum. It was then immersed into an oil bath thermostated at 70 ^{o}C to start the polymerization. After 9 h, the ampoule was quenched into liquid nitrogen to terminate the polymerization. The mixture was precipitated into an excess of diethyl ether to generate light pink residues, the residues were dissolved in DCM and precipitated into diethyl ether. The final product was dried in a vacuum oven overnight at room temperature, affording TPP-PDMA a light pink solid powder (654.9 mg). Following similar procedures, other polyprodrug amphiphiles were also synthesized. The structural parameters of all polyprodrug amphiphiles were summarized in Supplementary Table 1.

Conjugation of cRGD to the polyprodrug

Amine functionalized cRGD was conjugated to the PDMA-*b*-P(CPTSM-*co*-RhB) polyprodrug amphiphiles using an EDC/sulfo-NHS technique.[7] PDMA-*b*-P(CPTSM-*co*-RhB) (40 mg) were suspended in 2 mL anhydrous DCM with EDC (0.7 mg) and NHS (0.4 mg) at room temperature for 36 min. The NHS-activated PDMA-*b*-P(CPTSM-*co*-RhB) polyprodrug amphiphiles were allowed to react with amine-terminated cRGD (2 mg) for 24 h under magnetic stirring. The cRGD functionalized polyprodrug amphiphiles was precipitated into an excess of diethyl ether to generate red residues, the residues were dissolved in DCM and precipitated into diethyl ether. The final product was dried in a vacuum oven overnight at room temperature, yielding a red solid powder (39.5 mg).

Fabrication of dual-targeted polyprodrug nanoreactors

Typical self-assembly procedures of DT-PNs are as follows.[8] cRGD-PDMA-*b*-PCPTSM (1.0 mg) and TPP-PDMA-*b*-PCPTSM (1.0 mg) was first dissolved in 1 mL DMSO, and then quickly injected into 9 mL deionized water in one shot under vigorous stirring. The colloidal dispersion was further stirred, followed by

dialysis (Mw cutoff, 3.5 kDa) against deionized water overnight to remove DMSO. During this process, fresh deionized water was replaced. Following similar procedures, cRGD-PNs, TPP-PNs, and NT-PNs were also prepared. The final volume was adjusted to 1 mL by ultrafiltration (20,000 MWCO, Amicon, Millipore Corporation, Bedford, USA) for further experiments.

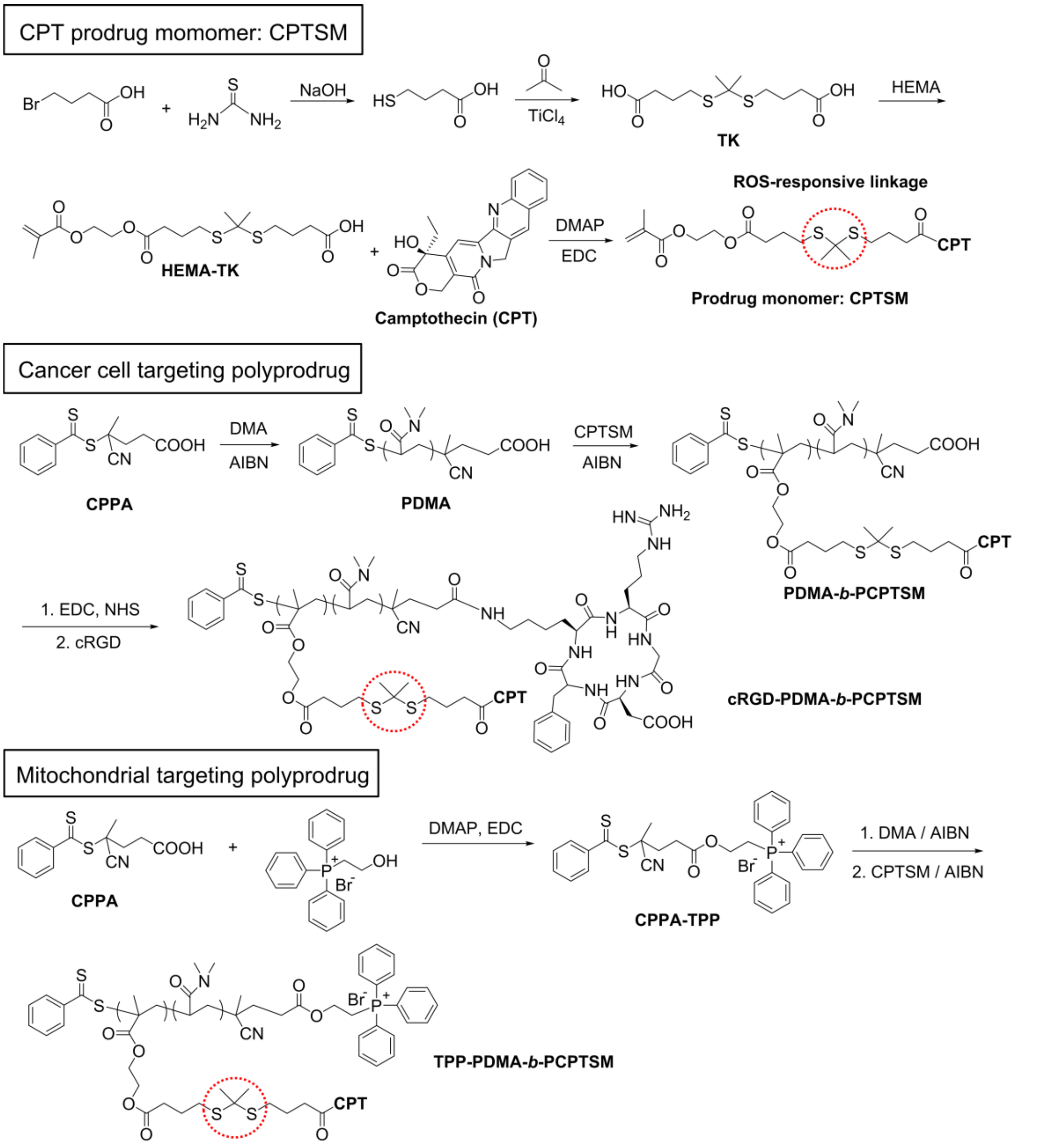

Supplementary Figure 1. Synthetic procedure employed for CPT prodrug monomer, CPTSM, and ROS-responsive polyprodrug amphiphiles with distinct targeting moieties.

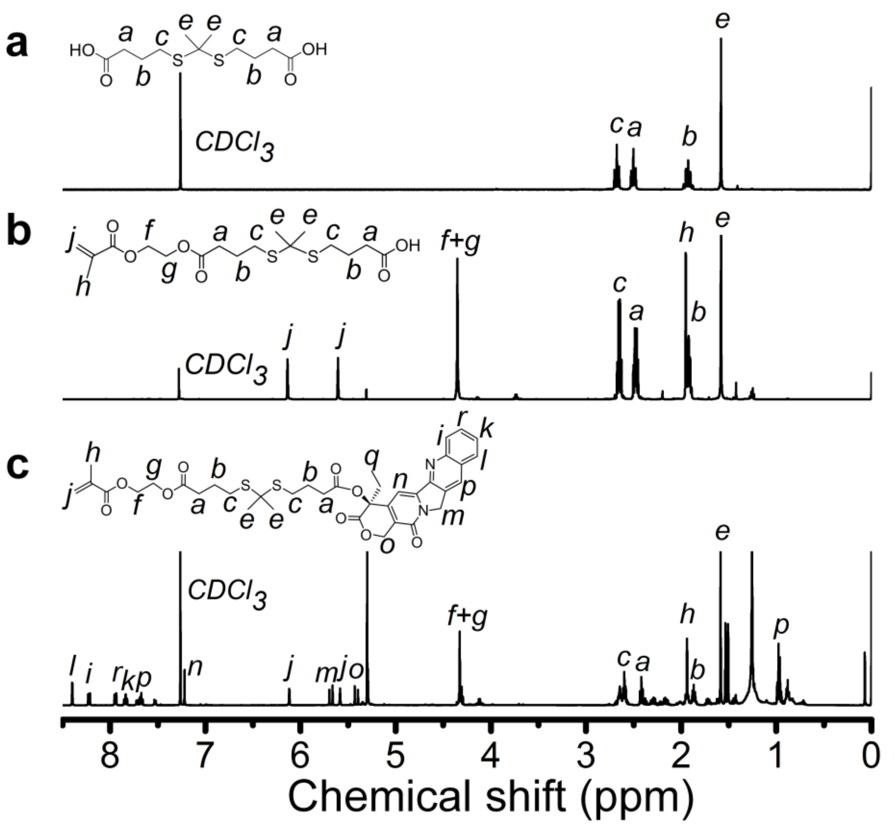

Supplementary Figure 2. ^{1}H NMR spectra recorded in $CDCl_3$ for the precursors and CPTSM. (a) TK, (b) HEMA-TK, (c) CPTSM.

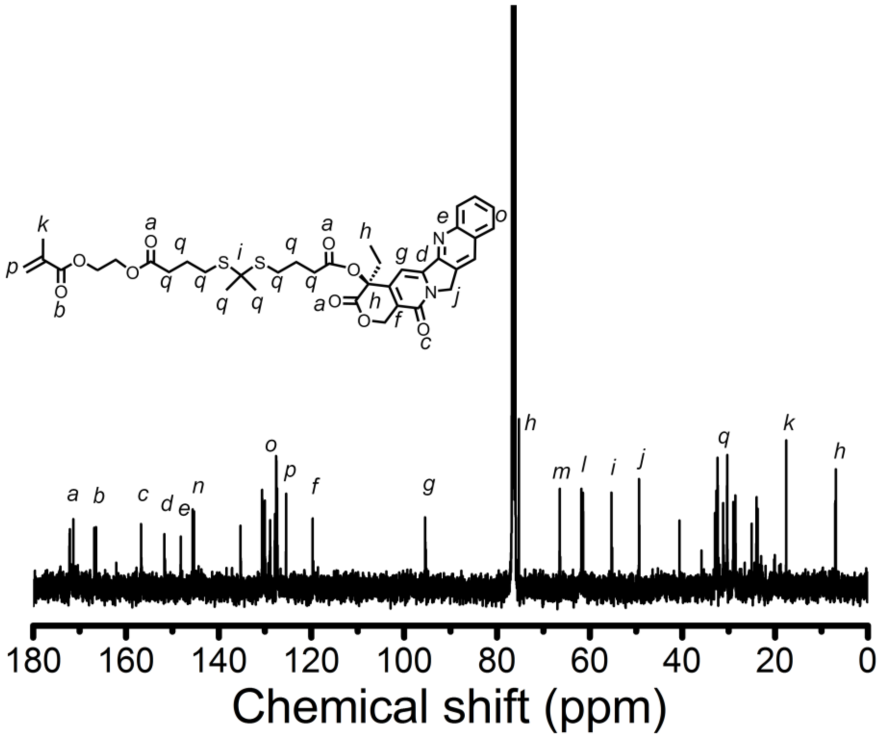

Supplementary Figure 3. ^{13}C NMR spectrum recorded for CPTSM in $CDCl_3$.

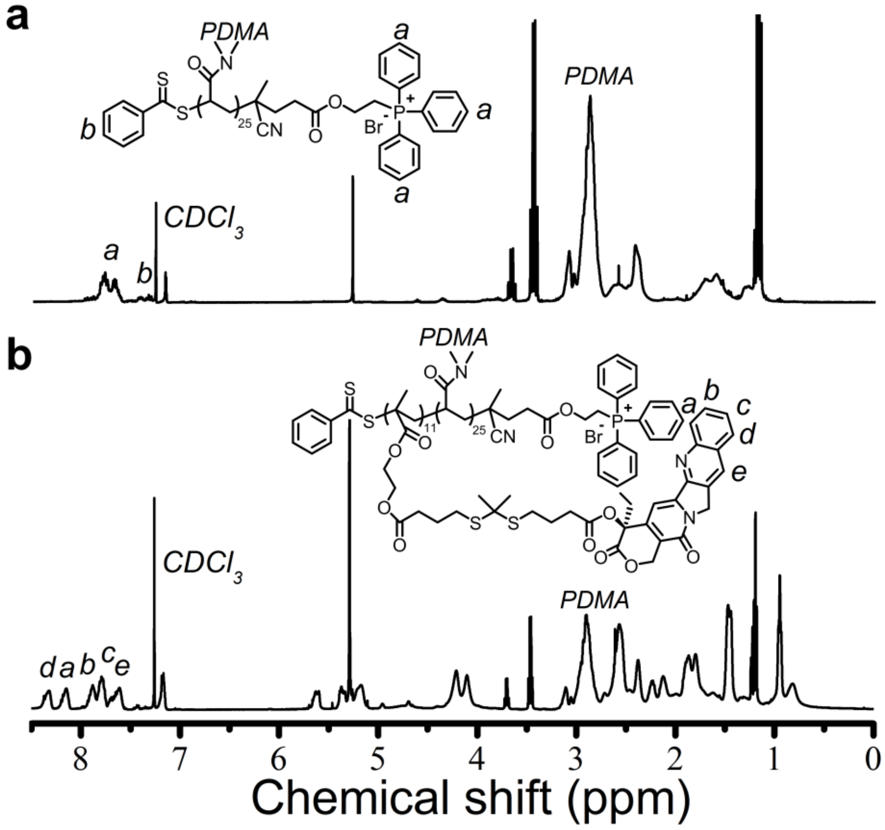

Supplementary Figure 4. ^{1}H NMR spectra recorded for mitochondria-targeting polyprodrug amphiphiles in $CDCl_3$. (a) $PDMA_{25}$, (b) TPP-PDMA-*b*-PCPTSM.

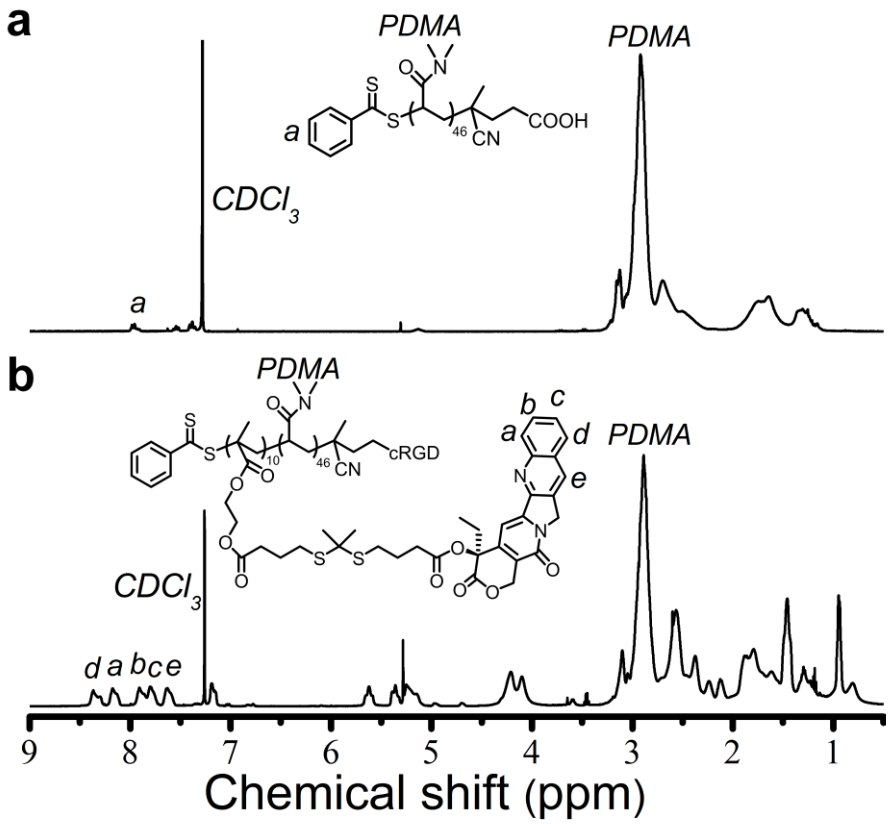

Supplementary Figure 5. ^{1}H NMR spectrum recorded for cancer cell targeting polyprodrug amphiphiles in $CDCl_3$. (a) $PDMA_{46}$, (b) cRGD-PDMA-*b*-PCPTSM.

Supplementary Table 1. Detailed Structural Parameters of Polyprodrug Amphiphiles.

**Polyprodrug Amphiphiles**	**DP**[a]	$\mathbf{M_{n,\ NMR}}$ **(kDa)**[a]	$\mathbf{M_{n,\ GPC}}$ **(kDa)**[b]	$\mathbf{M_w/M_n}$[b]	**DLC/ %**[c]
$PDMA_{25}$-*b*-$PCPTSM_{11}$	11	10.5	10.1	1.17	36.58
TPP-$PDMA_{25}$-*b*-$PCPTSM_{11}$	11	11.2	10.6	1.24	34.36
$PDMA_{46}$-*b*-P(CPTSM-*co*-$RhB_{0.02})_{10}$	10	12.2	10.3	1.23	28.59
cRGD-$PDMA_{46}$-*b*-P(CPTSM-*co*-$RhB_{0.02})_{10}$	10	12.8	10.9	1.19	27.24

[a]Number averaged molecular weights of polyprodrug amphiphiles and average degrees of polymerization (DPs) for the PCPTSM block were determined by ^{1}H NMR. [b]Molecular weights and molecular weight distributions, M_w/M_n, were evaluated by DMF GPC with polystyrene standards. [c]Drug loading content (DLC) was calculated as the weight ratio of conjugated drug to the weight of polyprodrug amphiphiles.

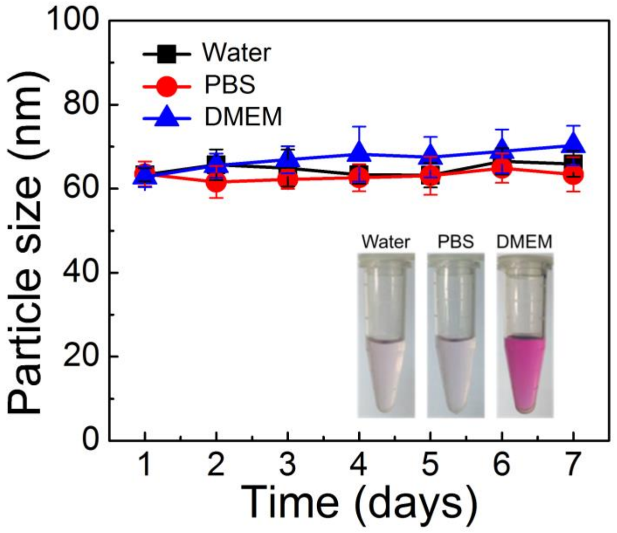

Supplementary Figure 6. Average diameter change of DT-PNs upon incubating in water, PBS, or DMEM containing 10 % fetal bovine serum (FBS) for 7 days. The inset shows photographs of samples after storing for 7 days at 37 ^{o}C.

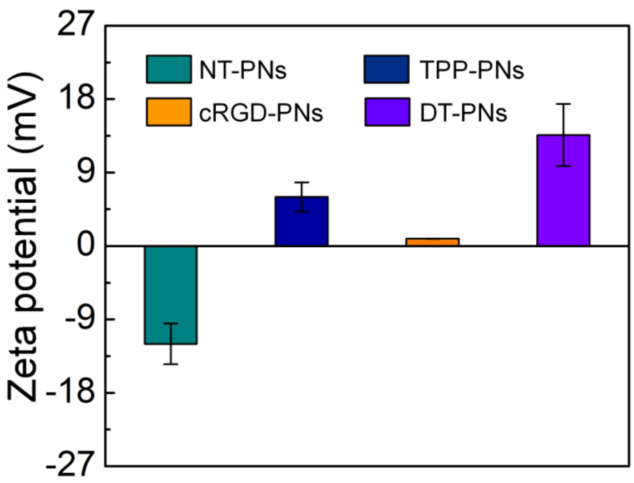

Supplementary Figure 7. Zeta potential determined for the dispersion of NT-PNs, TPP-PNs, cRGD-PNs and DT-PNs in PBS buffer.

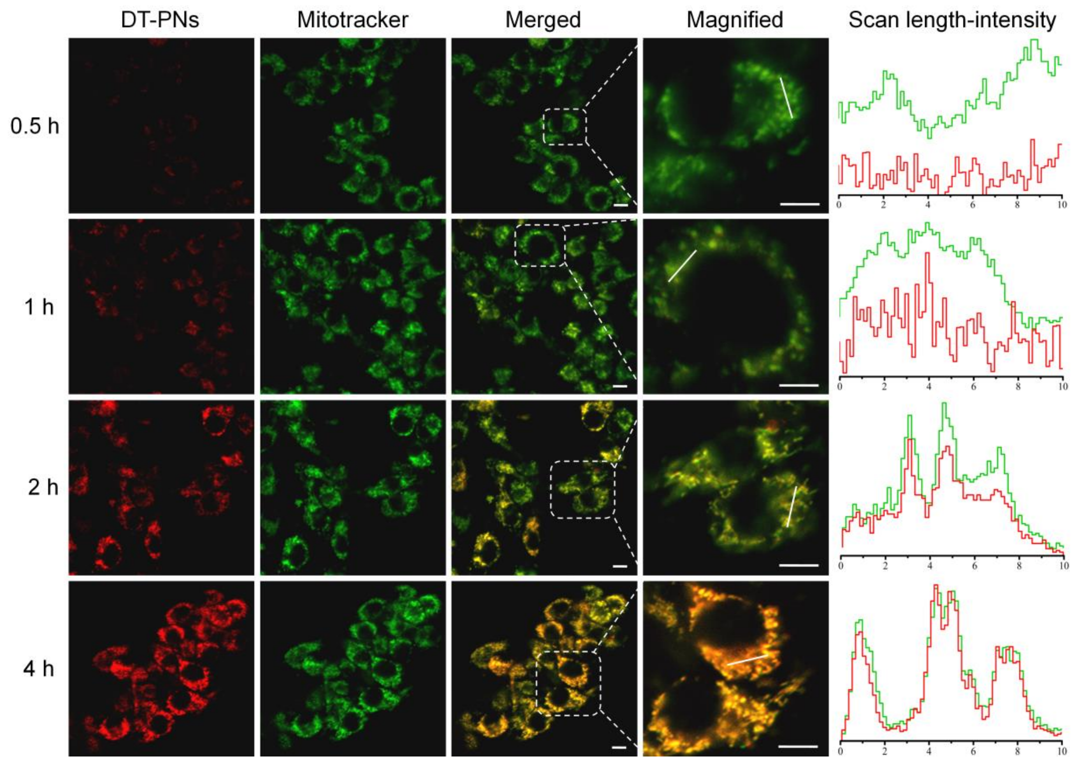

Supplementary Figure 8. Mitochondria targeting property of RhB-labelled DT-PNs against 4T1 cells. Mitochondria were stained with Mitotracker Green and noted as green. 4T1 cells were incubated with DT-PNs for different durations, scale bar: 20 μm.

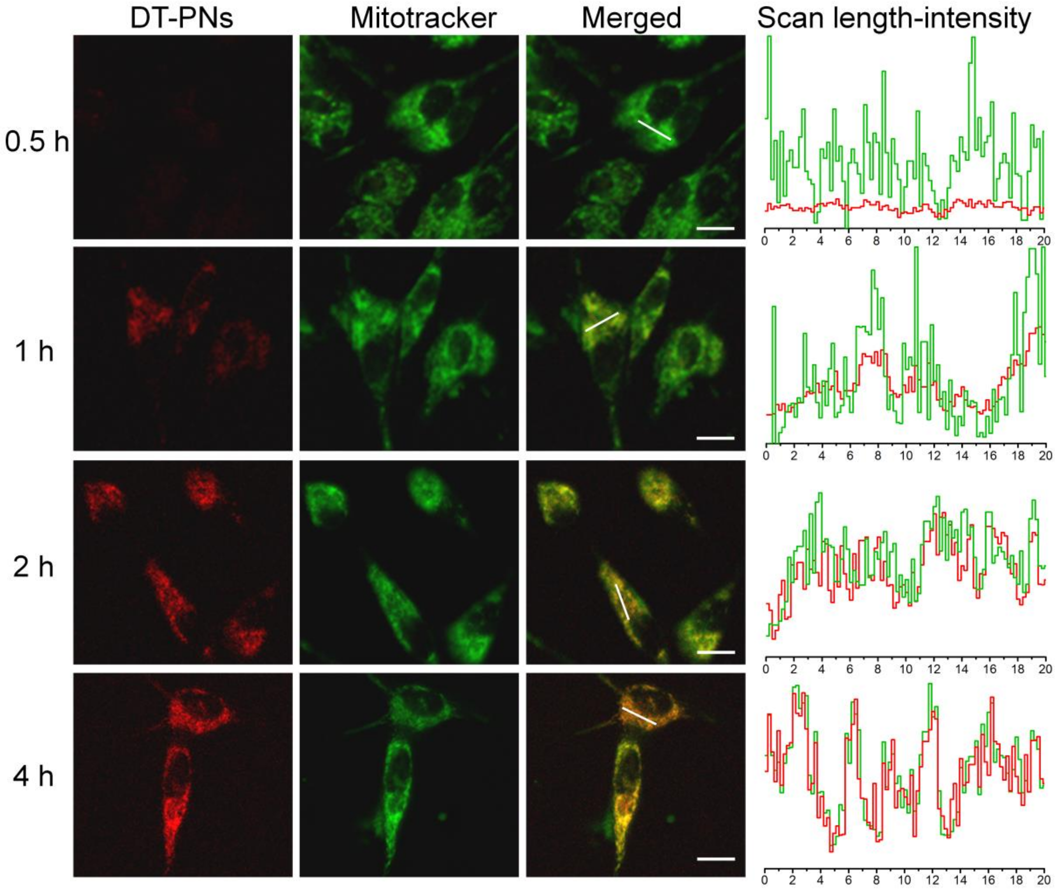

Supplementary Figure 9. Mitochondria targeting property of RhB-labelled DT-PNs against U87 cells. Mitochondria were stained with Mitotracker Green and noted as green. U87 cells were incubated with DT-PNs for different durations, scale bar: 20 μm.

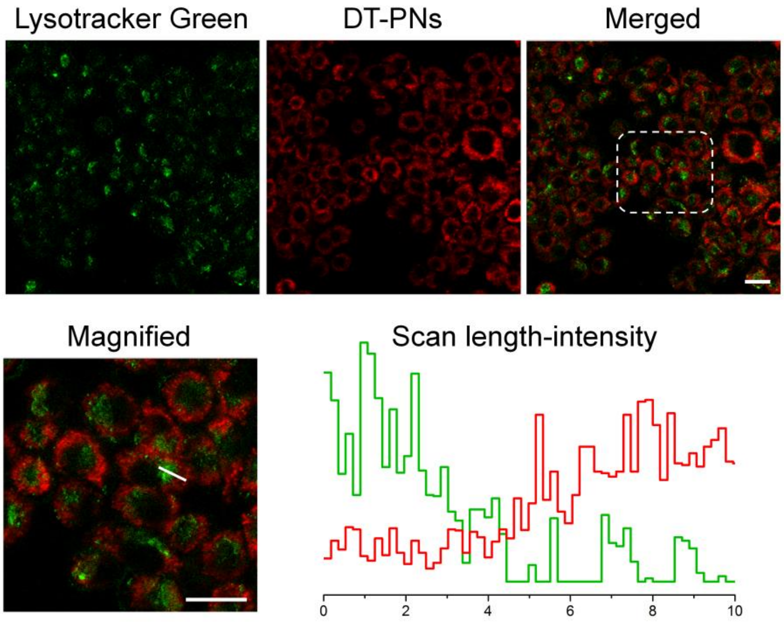

Supplementary Figure 10. Confocal images of 4T1 cells upon incubation with RhB-labelled DT-PNs for 4 h, the late endosomes and lysosomes were stained with Lysotracker Green, scale bar: 10 μm.

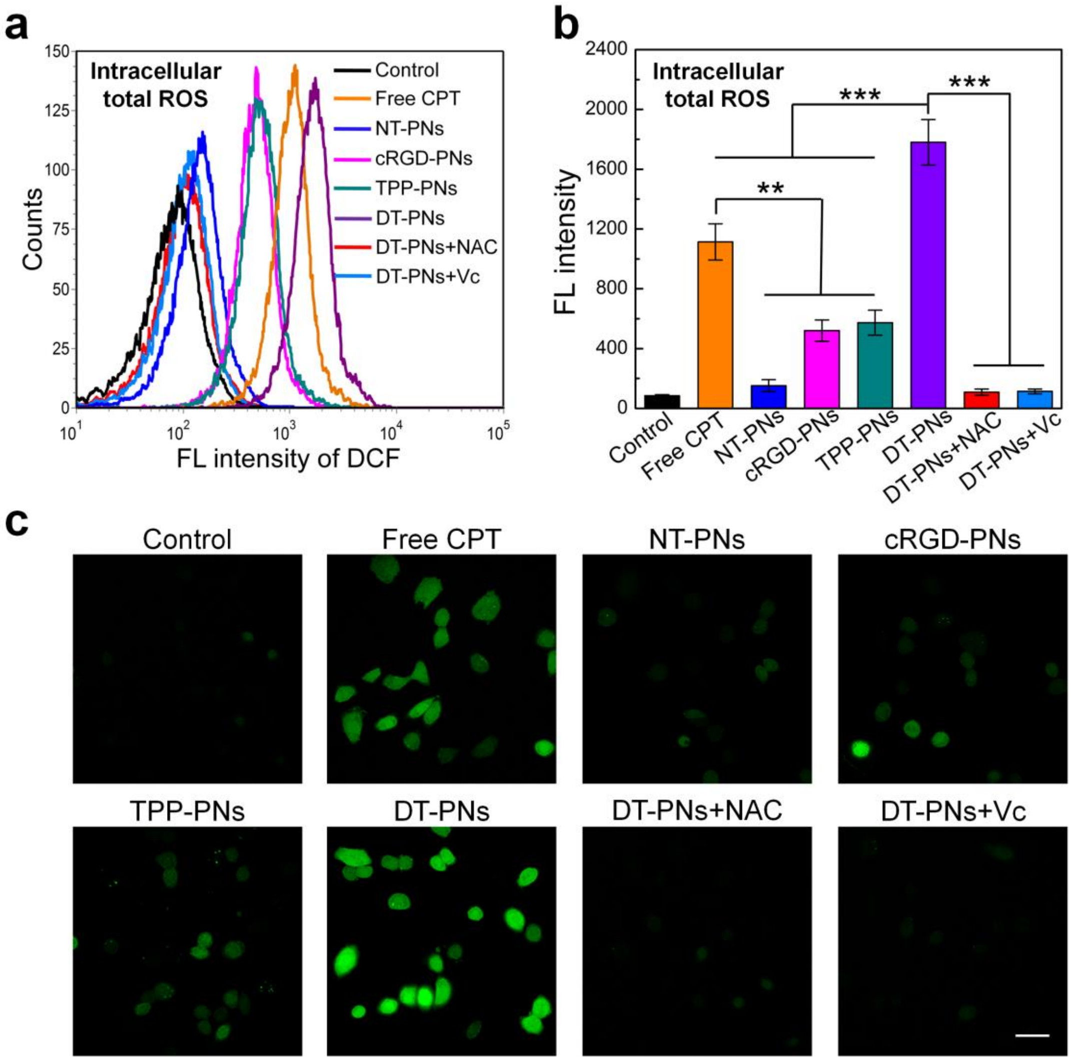

Supplementary Figure 11. Determination of intracellular total ROS level for 4T1 cells upon different treatments for 8 h detected by DCFH-DA based on flow cytometry analysis. (b) Statistical analysis of the mean fluorescence intensity in (a). The mean value was calculated by the *t* test (n=3). $**p < 0.01$, $***p < 0.001$, compared with the indicated group. (c) CLSM imaging of intracellular total ROS by DCFH-DA staining upon incubation with DMEM medium (control), free CPT, NT-PNs, cRGD-PNs, TPP-PNs, and DT-PNs with/without 20 mM NAC or 10 mM Vc for 8 h, respectively.

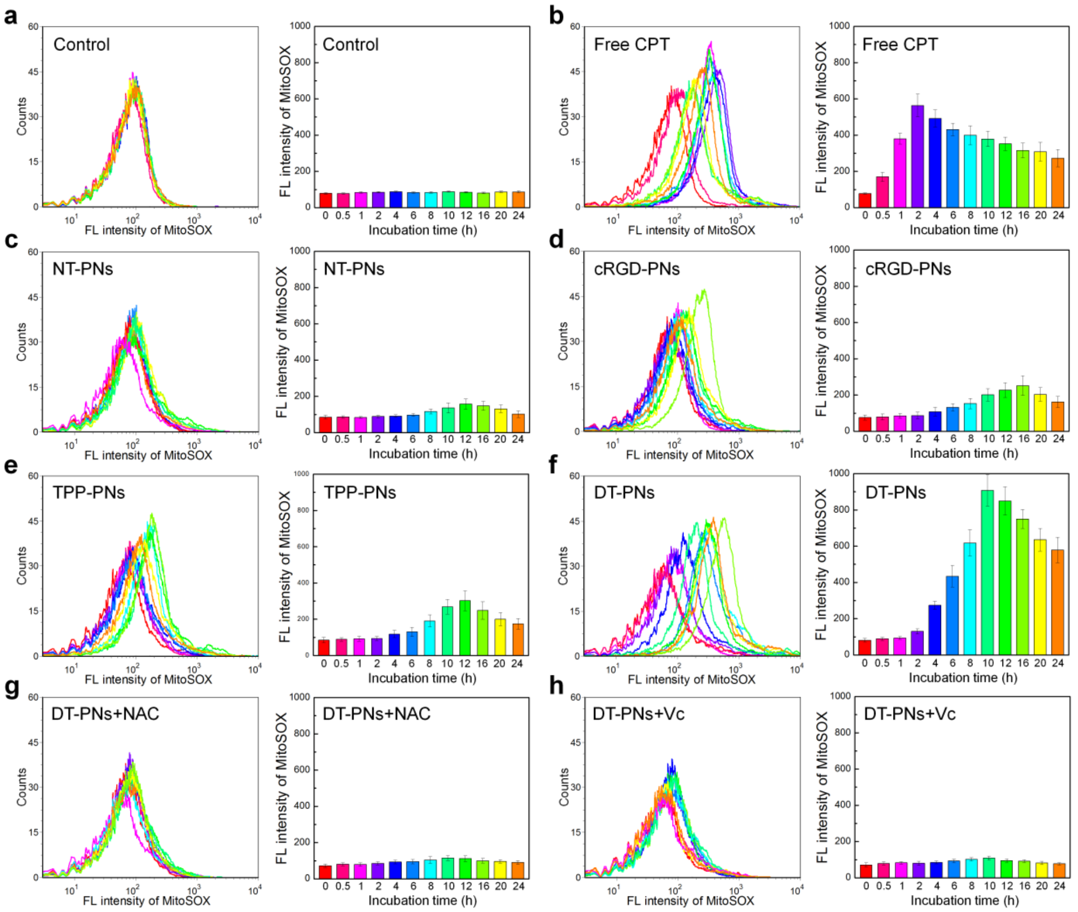

Supplementary Figure 12. Kinetic monitoring of mitochondrial superoxide by flow cytometry for 4T1 cells after diverse treatments within 24 h. (a) control, (b) free CPT, (c) NT-PNs, (d) cRGD-PNs, (e) TPP-PNs, (f) DT-PNs, (g) DT-PNs+NAC, (h) DT-PNs+Vc. MitoSOX Red was employed as a fluorescent indicator of mitochondrial superoxide.

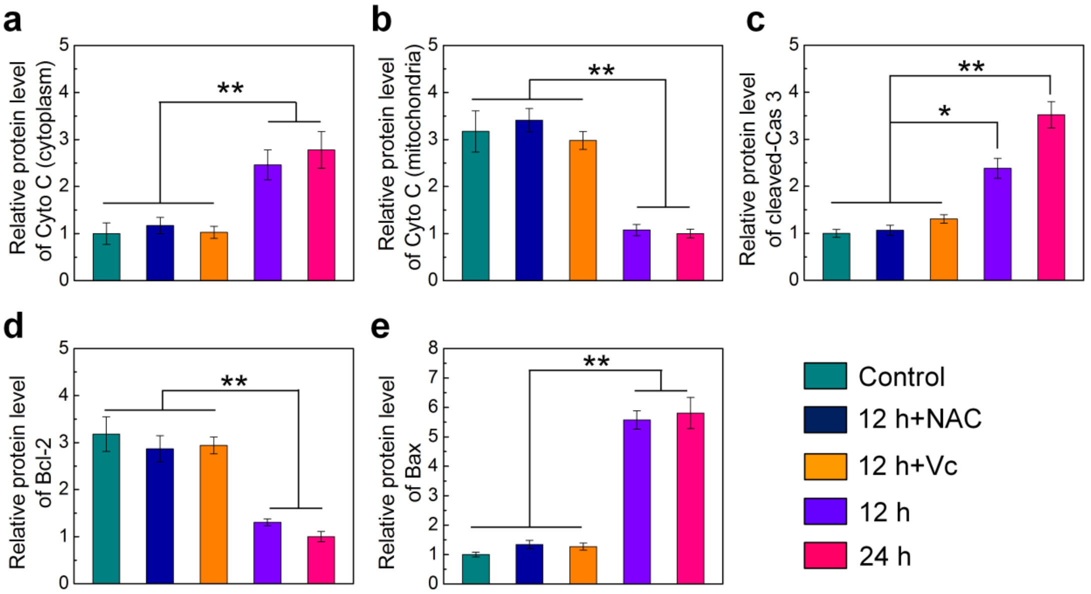

Supplementary Figure 13. Densitometry analysis of the amount of Cyto C, cleaved-caspase 3, Bcl-2 and Bax proteins after treated DT-PNs for 12 h and 24 h or with NAC and Vc prior to treatment. The sample notes refer to all panels (a)-(e). The mean value was calculated by the *t* test (n=3).*p < 0.05, **p < 0.01, versus the indicated group.

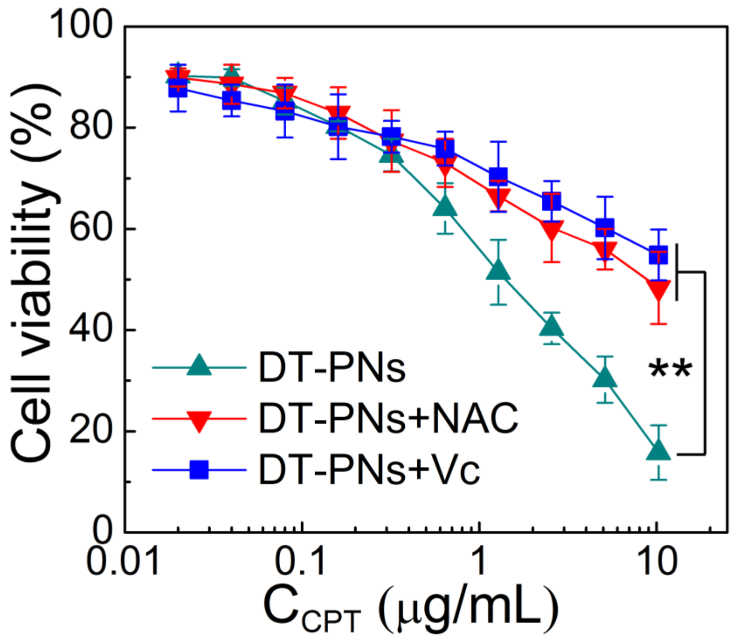

Supplementary Figure 14. *In vitro* cytotoxicity determined by MTT assay against 4T1 cells upon 36 h treatment with DT-PNs with or without NAC or Vc. The mean value was calculated by the *t* test (n=6). ***p* < 0.01, compared with the indicated group.

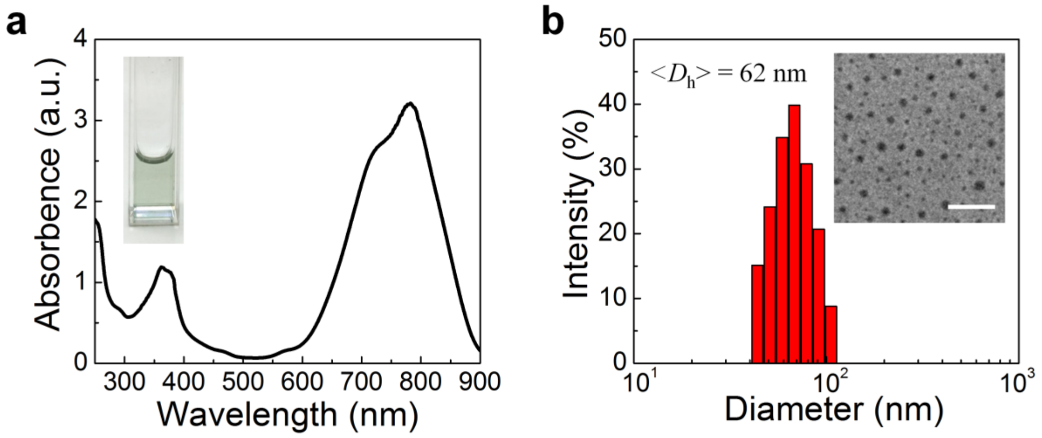

Supplementary Figure 15. (a) Absorption spectrum and (b) hydrodynamic diameter distribution (inset: TEM image) recorded for ICG-loaded dual-targeting polyprodrug nanoparticles, ICG@DT-PNs. Scale bar: 200 nm.

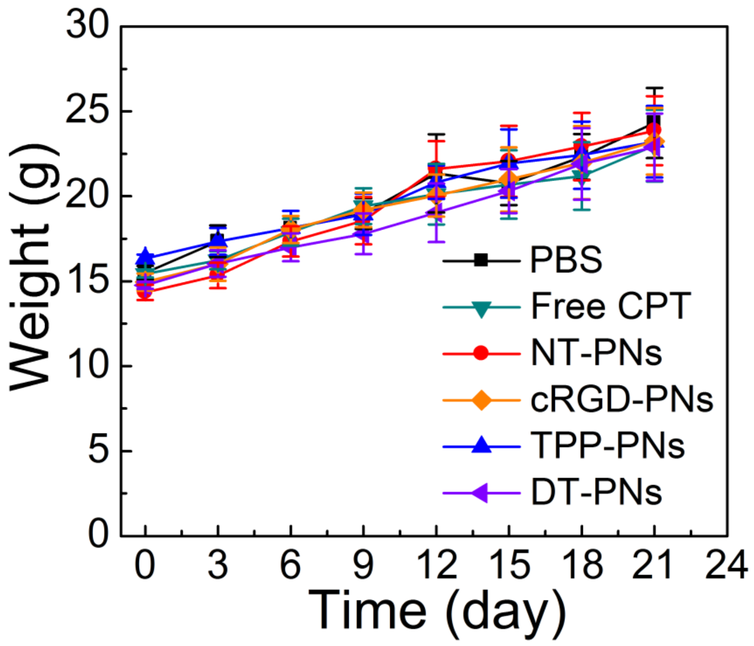

Supplementary Figure 16. The body weight of 4T1 tumor-bearing mice after different treatments. Data are represented as mean ±s.e.m. n = 6.

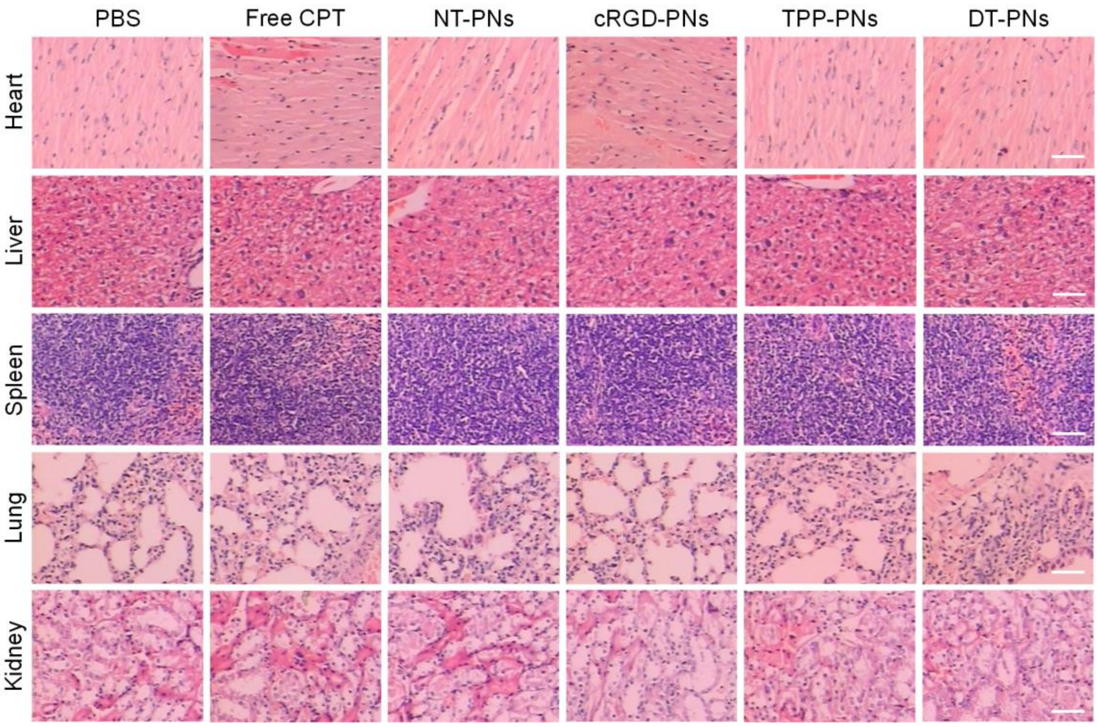

Supplementary Figure 17. Representative images of H&E stained heart, liver, spleen, lung and kidneys from the PBS, free CPT, NT-PNs, cRGD-PNs, TPP-PNs and DT-PNs treated group, respectively. Scar bar, 50 μm.

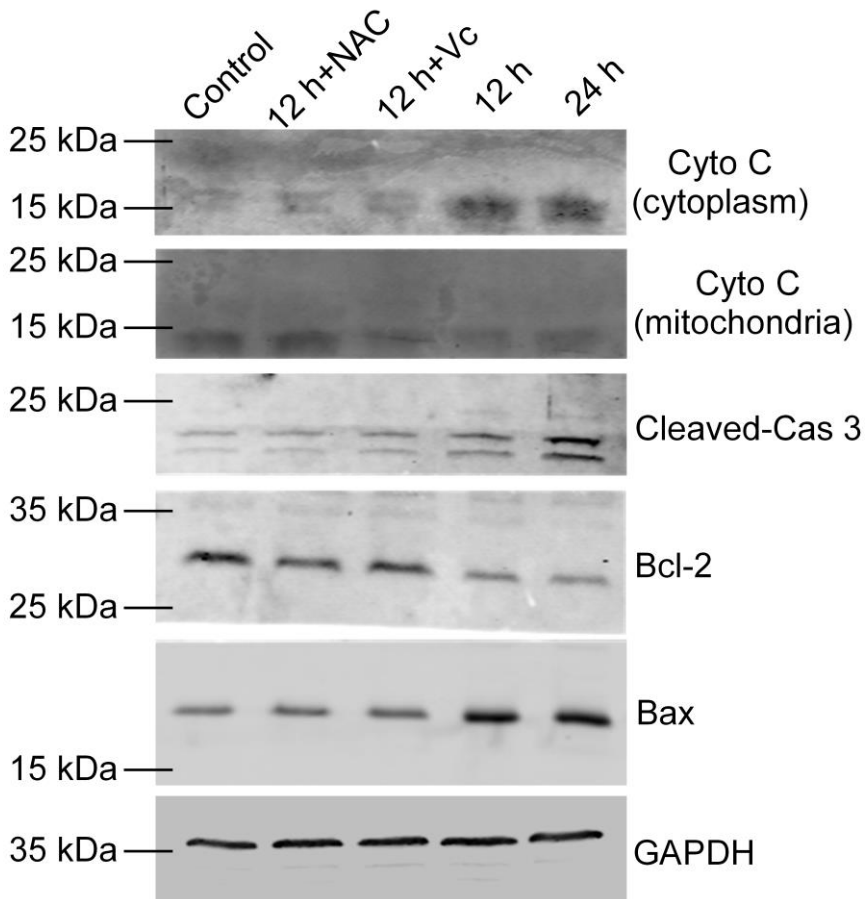

Supplementary Figure 18. Uncropped scans of western blot with molecular weight markers, it is related with Figure 6d.

References

1. Lin, Y. A. *et al.* Multiwalled nanotubes formed by catanionic mixtures of drug amphiphiles. *Acs Nano* **8**, 12690-12700 (2014).
2. Blount, K. F. & Uhlenbeck, O. C. Internal equilibrium of the hammerhead ribozyme is altered by the length of certain covalent cross-links. *Biochemistry* **41**, 6834-6841 (2002).
3. Devarie-Baez, N. O. *et al.* Light-induced hydrogen sulfide release from "caged" gem-dithiols. *Org. Lett.* **15**, 2786-2789 (2013).
4. Kim, I. H. *et al.* Optimization of amide-based inhibitors of soluble epoxide hydrolase with improved water solubility. *J. Med. Chem.* **48**, 3621-3629 (2005).
5. Hu, X. L., Liu, G. H., Li, Y., Wang, X. R. & Liu, S. Y. Cell-penetrating hyperbranched polyprodrug amphiphiles for synergistic reductive milieu-triggered drug release and enhanced magnetic resonance signals. *J. Am. Chem. Soc.* **137**, 362-368 (2015).
6. Zobrist, C. *et al.* Functionalization of titanium surfaces with polymer brushes prepared from a biomimetic raft agent. *Macromolecules* **44**, 5883-5892 (2011).
7. Schiffelers, R. M. *et al.* Cancer sirna therapy by tumor selective delivery with ligand-targeted sterically stabilized nanoparticle. *Nuclear Acids Res.* **32**, e149 (2004).
8. Hu, X. L. *et al.* Polyprodrug amphiphiles: Hierarchical assemblies for shape-regulated cellular internalization, trafficking, and drug delivery. *J. Am. Chem. Soc.* **135**, 17617-17629 (2013).
