# Supplementary material for: Mitochondria-specific drug release and reactive oxygen species burst induced by polyprodrug nanoreactors can enhance chemotherapy
Source: Nat Commun. 2019 Apr 12;10:1704. doi: 10.1038/s41467-019-09566-3 (PMC6461692; doi:10.1038/s41467-019-09566-3)
Supplement: Supplementary file 3 — Reporting Summary [file 41467_2019_9566_MOESM3_ESM.pdf]

nature research

Corresponding author(s): Da Xing, Xianglong Hu

Last updated by author(s): Feb. 14, 2019

Reporting Summary

Nature Research wishes to improve the reproducibility of the work that we publish. This form provides structure for consistency and transparency in reporting. For further information on Nature Research policies, see Authors & Referees and the Editorial Policy Checklist.

Statistics

For all statistical analyses, confirm that the following items are present in the figure legend, table legend, main text, or Methods section.

n/a	Confirmed	
☐	☒	The exact sample size (*n*) for each experimental group/condition, given as a discrete number and unit of measurement
☐	☒	A statement on whether measurements were taken from distinct samples or whether the same sample was measured repeatedly
☐	☒	The statistical test(s) used AND whether they are one- or two-sided<br>*Only common tests should be described solely by name; describe more complex techniques in the Methods section.*
☐	☒	A description of all covariates tested
☐	☒	A description of any assumptions or corrections, such as tests of normality and adjustment for multiple comparisons
☐	☒	A full description of the statistical parameters including central tendency (e.g. means) or other basic estimates (e.g. regression coefficient) AND variation (e.g. standard deviation) or associated estimates of uncertainty (e.g. confidence intervals)
☐	☒	For null hypothesis testing, the test statistic (e.g. *F*, *t*, *r*) with confidence intervals, effect sizes, degrees of freedom and *P* value noted<br>*Give P values as exact values whenever suitable.*
☒	☐	For Bayesian analysis, information on the choice of priors and Markov chain Monte Carlo settings
☒	☐	For hierarchical and complex designs, identification of the appropriate level for tests and full reporting of outcomes
☒	☐	Estimates of effect sizes (e.g. Cohen's *d*, Pearson's *r*), indicating how they were calculated

Our web collection on statistics for biologists contains articles on many of the points above.

Software and code

Policy information about availability of computer code

Data collection	Thanks, no software was used.
Data analysis	Origin 8, Image J software, ChemDraw

For manuscripts utilizing custom algorithms or software that are central to the research but not yet described in published literature, software must be made available to editors/reviewers. We strongly encourage code deposition in a community repository (e.g. GitHub). See the Nature Research guidelines for submitting code & software for further information.

Data

Policy information about availability of data

All manuscripts must include a data availability statement. This statement should provide the following information, where applicable:

- Accession codes, unique identifiers, or web links for publicly available datasets
- A list of figures that have associated raw data
- A description of any restrictions on data availability

Data availability. The data in this work are available from the corresponding author upon request

Field-specific reporting

Please select the one below that is the best fit for your research. If you are not sure, read the appropriate sections before making your selection.

☒ Life sciences ☐ Behavioural & social sciences ☐ Ecological, evolutionary & environmental sciences

For a reference copy of the document with all sections, see nature.com/documents/nr-reporting-summary-flat.pdf

Life sciences study design

All studies must disclose on these points even when the disclosure is negative.

Sample size	Generally accepted sample sizes were used. Sample size was determined based on standard practices and our previous experience. The number of independent experiment and replicates was shown in the corresponding figure legend.
Data exclusions	No data exclusion.
Replication	All the experiments were reliably reproduced.
Randomization	Randomization was employed whenever it was deemed necessary.
Blinding	In most of the experiments, the investigators were not blinded to group allocation, as the experimental observations would be consistent irrespective of blinding. Conclusions were made based on independent experiments, quantitative parameters and statistical significance of the data. Occasionally blinding was deployed, as in tumor size measurement, with the measuring person having no knowledge on the identity of the mice being measured.

Reporting for specific materials, systems and methods

We require information from authors about some types of materials, experimental systems and methods used in many studies. Here, indicate whether each material, system or method listed is relevant to your study. If you are not sure if a list item applies to your research, read the appropriate section before selecting a response.

Materials & experimental systems

n/a	Involved in the study	
☐	☒	Antibodies
☐	☒	Eukaryotic cell lines
☒	☐	Palaeontology
☐	☒	Animals and other organisms
☒	☐	Human research participants
☒	☐	Clinical data

Methods

n/a	Involved in the study	
☒	☐	ChIP-seq
☐	☒	Flow cytometry
☒	☐	MRI-based neuroimaging

Antibodies

Antibodies used	The following antibodies were used for immunoblot: rabbit monoclonal anti-cytochrome c antibody (1:1000 dilution, Cell Signaling Technology), rabbit monoclonal anti-caspase-3 antibody (1:1000 dilution, Cell Signaling Technology), mouse monoclonal anti-Bcl-2 antibody (1:1000 dilution, Cell Signaling Technology), mouse monoclonal anti-Bax antibody (1:1000 dilution, Cell Signaling Technology) and moue monoclonal GAPDH antibody (1:1000 dilution, Cell Signaling Technology), IRDye 800 CW anti-rabbit IgG (1:3000 dilution, Santa Cruz Biotechnoogy), Alexa Fluor 680 goat anti-Mouse IgG (1:3000 dilution, Santa Cruz Biotechnoogy).
Validation	All antibodies are from commercial sources and their validation data are available on the manufacturer's website.

Eukaryotic cell lines

Policy information about cell lines

Cell line source(s)	4T1 rat breast cancer cells , MCF-7 human breast cancer cells and U87 human glioblastoma cells were purchased from ATCC.
Authentication	Authentication was performed by ATCC for three kinds of cell lines.
Mycoplasma contamination	All of three types of cell lines tested negative for mycoplasma contamination.
Commonly misidentified lines (See ICLAC register)	The cell lines used in the study are not listed commonly misidentified cell lines in the ICLAC list.

Animals and other organisms

Policy information about studies involving animals; ARRIVE guidelines recommended for reporting animal research

Laboratory animals	Female Balb/c mice (five weeks old) were purchased from Center for Experimental Animals, Southern Medical University, and housed in the SPF facility.

Wild animals

No wild animals involved in this study.

Field-collected samples

The study did not involve samples collected from the field.

Ethics oversight

All animal studies were conducted in accordance with the guidelines of the National Regulation of China for Care and Use of Laboratory Animals (South China Normal University, Guangzhou, China).

Note that full information on the approval of the study protocol must also be provided in the manuscript.

Flow Cytometry

Plots

Confirm that:

☒ The axis labels state the marker and fluorochrome used (e.g. CD4-FITC).

☒ The axis scales are clearly visible. Include numbers along axes only for bottom left plot of group (a 'group' is an analysis of identical markers).

☒ All plots are contour plots with outliers or pseudocolor plots.

☒ A numerical value for number of cells or percentage (with statistics) is provided.

Methodology

Sample preparation

4T1 cells were seeded on 6-well plates at a density of 100000, respectively. After incubation for 24 h, the cells were treated with various samples and incubated for 6 h at 37 °C. Then, the cells were washed with cold PBS, harvested and analyzed immediately using flow cytometry. The mean fluorescence intensity of 10000 cells was recorded for each sample.

Instrument

Cytomics FC 500, Beckman Coulter, USA

Software

FCS Express

Cell population abundance

Not applicable.

Gating strategy

Compensation was used wherever necessary.

☒ Tick this box to confirm that a figure exemplifying the gating strategy is provided in the Supplementary Information.
